# Mangiferin‐Encapsulated Lipid Carriers: Innovative strategy for Enhanced Brain Delivery in Alzheimer's Therapy

**DOI:** 10.1002/alz70861_108327

**Published:** 2025-12-23

**Authors:** Akshatha P Kamath, Nandakumar Krishnadas, Pawan G Nayak

**Affiliations:** ^1^ Manipal College of Pharmaceutical Sciences, Manipal Academy of Higher Education, India, Udupi, Karnataka India; ^2^ Manipal College of Pharmaceutical Sciences, Manipal Academy Of Higher Education, Manipal, Karnataka India

## Abstract

**Background:**

Alzheimer's disease (AD) is a neurodegenerative disease involving amyloid‐β plaques and tau tangles leading to cognitive impairment. Mangiferin (MGF), a xanthone with neuroprotective activity, has low bioavailability. Lipid nanoparticles enhance brain targeting and solubility of MGF. This study aims to develop Mangiferin‐Lipid Carriers and determine their therapeutic efficacy in AD‐induced rats.

**Method:**

Pre‐formulation studies to identify suitable excipients for lipid nanoparticle preparation. The formulation was optimized using Design of Experiments (DoE) software to achieve better solubility and stability. Characterization included particle size analysis, zeta potential measurement, drug loading, and encapsulation efficiency. Reverse‐phase HPLC (RP‐HPLC) was used for analytical method validation and quantification. Structural integrity was assessed using transmission electron microscopy (TEM) and differential scanning calorimetry (DSC).

**Result:**

Pre‐formulation studies identified compritol 888 ATO, soy lecithin, and tween 80 as excipients for MGF‐loaded LCs. The optimized formulation possessed a particle size of 170–210nm, zeta potential of ‐29mV, and 73% entrapment efficiency without any aggregation. RP‐HPLC validation showed Rt of 7.1min, NTP of 2953.87, tf of 1.2, and peak area of 699708mV‐min. It was well linear with R² = 0.9999 and LOQ and LOD of 1.56 and 0.52ng/mL, respectively. TEM and DSC analysis also confirmed the structural integrity and morphology of the MGF‐LCs.

**Conclusion:**

MGF‐LCs demonstrated ideal properties for brain targeting, including nanoscale size, high Entrapment, and stability. RP‐HPLC validated accurate quantification. These findings suggest that MGF‐LCs can enhance bioavailability and serve as a promising intervention for AD. Further in vivo studies are essential to assess their neuroprotective efficacy and confirm their therapeutic potential.

